# Systematic evaluation of genome-wide methylated DNA enrichment using a CpG island array

**DOI:** 10.1186/1471-2164-12-10

**Published:** 2011-01-06

**Authors:** Liu Yang, Kunlin Zhang, Wei Dai, Ximiao He, Qian Zhao, Jing Wang, Zhong Sheng Sun

**Affiliations:** 1 Key Laboratory of Mental Health, Institute of Psychology, Chinese Academy of Sciences, 100101 Beijing, PR China; 2 Behavioral Genetics Center, Institute of Psychology, Chinese Academy of Sciences, 100101 Beijing, PR China; 3 Zhejiang University School of Medicine, 310058 Hangzhou, PR China

## Abstract

**Background:**

Recent progress in high-throughput technologies has greatly contributed to the development of DNA methylation profiling. Although there are several reports that describe methylome detection of whole genome bisulfite sequencing, the high cost and heavy demand on bioinformatics analysis prevents its extensive application. Thus, current strategies for the study of mammalian DNA methylomes is still based primarily on genome-wide methylated DNA enrichment combined with DNA microarray detection or sequencing. Methylated DNA enrichment is a key step in a microarray based genome-wide methylation profiling study, and even for future high-throughput sequencing based methylome analysis.

**Results:**

In order to evaluate the sensitivity and accuracy of methylated DNA enrichment, we investigated and optimized a number of important parameters to improve the performance of several enrichment assays, including differential methylation hybridization (DMH), microarray-based methylation assessment of single samples (MMASS), and methylated DNA immunoprecipitation (MeDIP). With advantages and disadvantages unique to each approach, we found that assays based on methylation-sensitive enzyme digestion and those based on immunoprecipitation detected different methylated DNA fragments, indicating that they are complementary in their relative ability to detect methylation differences.

**Conclusions:**

Our study provides the first comprehensive evaluation for widely used methodologies for methylated DNA enrichment, and could be helpful for developing a cost effective approach for DNA methylation profiling.

## Background

The most widely studied epigenetic modification in humans is cytosine methylation at CpG dinucleotides. Computational analysis predictions have indicated there are about 29,000 CpG islands in the human genome [1,2]. Approximately 70% of CpG dinucleotides in mammals are methylated and found in repetitive elements [3] whereas most CpG islands with relative high densities of unmehylated CpG dinucleotides are located at the promoter region of house-keeping genes and tumor suppressor genes and play important roles in gene expression regulation and cellular differentiation [4]. Additionally, researchers have found that DNA methylation changes occur in human cancers [5], and researches in this area have established that hypermethylation of CpG islands tends to silence tumor suppressor genes and that hypomethylation activates oncogenes [6-8].

Many approaches for detecting DNA methylation are done in a gene-specific manner, such as bisulfite treatment of DNA combined with sequencing, amplification by methylation-specific PCR, or restriction enzyme-based digestion. These techniques are limited to study known candidate genes. Recent advances in DNA sequencing methods have now allowed genome-wide DNA methylation studies. However, even with the use of cost-effective next-generation sequencing technologies to carry out these analyses [9-11], there is still heavy reliance on high cost and high computational load of bioinformatics analyses, making sequencing methods still of limited application. Alternative genome-wide DNA methylation methods based on microarray technologies have proven to be useful. Additionally, due to the methylation patterns of the human genome described above, inclusion of a methylation enrichment approach can be extremely useful for detecting genome-wide DNA methylation patterns and changes.

The most popular genome-wide methylated DNA enrichment studies include approaches based on methylation-sensitive restriction-enzyme digestion. These include the following: differential methylation hybridization (DMH), which is a method for comparing the methylation status of CpG islands between test samples and control samples [12-14] and are widely used [15-22]. Microarray-based methylation assessment of single samples (MMASS), which utilizes methylation-sensitive and methylation-dependent enzyme digestion for within-sample comparison of methylation level of CpG island (CGI) loci [23]. Affinity purification by methylcytosine DNA-binding domain (MBD) protein, which uses an MBD-domain-conjugated column to purify methylated DNA fragments for DNA methylation assessment [24]. Immunoprecipitation of DNA using an antibody that recognizes 5'-methyl cytosine (MeDIP) [25-29], which was demonstrated to be more sensitive than MBD purification for detecting methylated DNA [30]. More approaches have also been developed recently [31,32].

Although the above global methylated DNA enrichment assays have demonstrated widespread utility, a systematic analysis of the sensitivity and accuracy of each of these assays has not been performed. In addition, within each method there is considerable variation in the use of each of the experimental parameters, which are important for enhancing the performance and many of them have not been adequately explored. Thus, a systematic evaluation of different approaches for genome-wide methylated DNA enrichment with optimized experimental parameters is necessary.

In this study, we set about to optimize several of the experimental parameters in these methodologies, and then we followed this up by performing a direct comparison between DMH, MMASS, and MeDIP. We additionally assessed potential reasons that underlie the variability in these assays. Our work provides the first results for evaluating these widely used enrichment assays, which will be useful for accurately analyzing the methylome in the epigenomics field.

## Results

### Evaluation of the quality of Human 9 K CGI array

As a first step for our analyses, we assessed the quality of our human 9 K CGI array (see Methods for array construction) as well as the consistency of our labeling. We hybridized two independently labeled aliquots (2 ug each) of sonicated genomic DNA with Cy5 and Cy3 fluorescent dyes, respectively. An MA plot of background-corrected and normalized log_2 _signal versus log_2 _differential signal from both the Cy5 and Cy3 channels is shown in Additional file 1, **Figure S1-A**. The signal consistency of two channels was very high with >99.5% of CGI probes showing <2-fold differential expression (|M|<1), and the signal from the two channels manifesting a strong correlation (Pearson correlation coefficient = 0.9975), confirming that our array was of high quality for use in the following experiments.

### Optimization of experimental parameters in DMH, MMASS, and MeDIP

DMH, MMASS, and MeDIP all have several parameters that can impact the quality of the results. To provide an assessment of the importance of each parameter and their impact on the experimental results, we investigated and optimized the primary parameters that could impact the results in these analyses. For DMH and MMASS assays, where the digested DNA products are amplified using PCR, the impact of annealing temperature in the PCR amplification of the digested products was assessed, and for MeDIP we looked at the incubation time of the 5-methylcytosine antibodies and secondary antibody during immunoprecipitation.

Methylation profiling of the gastric adenocarcinoma cell line MGC-803 was carried out using our Human 9 K CGI Array. The DNA products, derived from methylation sensitive restriction enzymes BstUI and HpaII (combined and called v1), and the methylation-dependent enzyme McrBC-digestion respectively, were PCR-amplified using three different annealing temperatures, i.e. 65ºC [23], 68.5°C (average of 65°C and 72°C), and 72°C [12,19]. We then hybridized equal amounts of the probe on the CGI array for within-sample comparison of methylation levels. Our results revealed that PCR-amplification at different annealing temperatures produced different methylation patterns after hybridization. Additional file 1, **Figure S1-B **shows that the array with an annealing temperature of 72°C had a higher total intensity and than the other two. Given this temperature providing the strongest signal, all the following DMH and MMASS experiments used 72°C as the optimal temperature for PCR amplification.

As antibody incubation time played a crucial role in the binding efficiency and specificity of methylated DNA fragments, To assess the specificity and efficiency of the methylated DNA fragment in MeDIP, we utilized two sets of external DNA controls from yeast genomic DNA, which had no significant homology with human and mouse genomic DNA sequence by BLAST (e value > 10^-3^), and each of the external DNA control was composed of methylated and unmethylated DNA fragments with a set ratio (Additional file 1, **Table S1, S2**) for assessment of optimized incubation time of anti-5-methylcytosine Mouse mAb and sheep anti-mouse IgG using a microarray-based methylation profiling study. The first tested condition employed anti-5-methylcytosine Mouse mAb with a 2 hr incubation time followed by incubation with sheep anti-mouse IgG for 2 hrs, as described previously [25]. The results showed that the hybridization ratio of IP compared to input of all the controls was much lower than the theoretical ratio, indicating that the methylated DNA binding with anti-5-methylcytosine Mouse mAb antibody was incomplete (Additional file 1, **Figure S2 (Top)**). We then increased the time of anti-5-methylcytosine Mouse mAb incubation to 12 hrs and found that the binding efficiency of external controls increased but still remained lower than the theoretical ratio shown in Additional file 1, **Figure S2 (Top)**.

Next the impact of the incubation time of the secondary antibody sheep anti-mouse IgG at 1 hr, 2 hrs, 4 hrs, and 6 hrs, was investigated respectively. The results showed the captured amount of methylated DNA increased with longer incubation time of the secondary antibody. However, nonspecific binding of unmethylated DNA fragments also rose (Additional file 1, **Figure S2 (middle)**). Nevertheless, the growth tendency showed the deviation of each external control from theoretical ratio was lowest around 3 hours. The results demonstrated that the optimal conditions for methylated DNA enrichment in MeDIP was a 12-hr incubation with the primary antibody followed by a 3-hr incubation with the secondary antibody. These optimized conditions were used in all following MeDIP experiments.

We also used our DNA external controls to evaluate the deviation of methylated DNA enrichment among DMH-v2, MMASS-v2, and MeDIP (using a 12-hr primary antibody incubation, and 2 hr, 3 hr, and 4 hr secondary antibody incubation). Our data showed that the deviation from the theoretical ratio of all external DNA controls was lowest for MeDIP (Additional file 1, **Figure S2 (Bottom)**). This indicates that whole genome amplification after digestion in DMH and MMASS may introduce PCR bias; thus, it is important when using those two assays to take this into consideration for evaluating results.

### Systematic comparison of DMH, MMASS, and MeDIP

After optimization of the above parameters for better performance for all three methods, a systematic evaluation of DMH, MMASS, and MeDIP was carried out using human 9 K CpG microarray for detecting differential methylation profiling of the gastric epithelium cell line Ges-1 and the gastric adenocarcinoma cell line MGC-803.

We first compared DMH and MMASS, both of which are based on restriction enzyme digestion. DMH typically employed methylation-sensitive enzyme digestion to enrich the methylated DNA fraction for between-samples comparison [12,16,18-20], whereas, MMASS used methylation-sensitive and methylation-dependent enzymes digestion for within-sample comparison [23,33].

**Figure 1:**
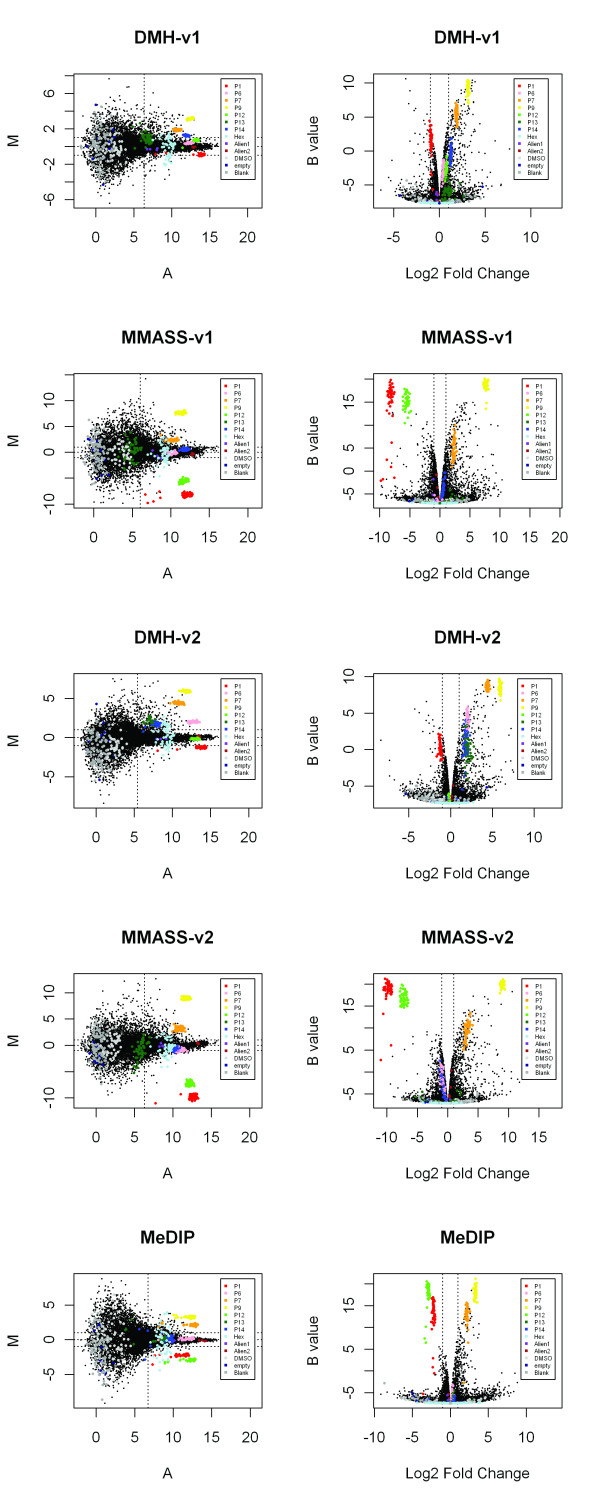
**MA plots and volcano plots showing data from the linear model fitted to replicate arrays for each of the DMH-v1, DMH-v2, MMASS-v1, MMASS-v2, and MeDIP methods**. Colored probes represent external DNA controls, Hex, Alien PCR product, DMSO, empty, and blank, respectively. The results of external DNA controls are nearly consistent with theoretical ratios. Plots of MMASS with either v1 or v2 set of enzymes show higher M, *B *values and Log_2_fold change than DMH and MeDIP methods.

**Figure 2:**
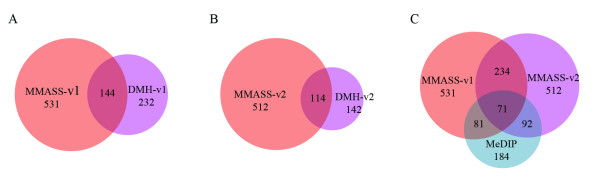
**Uniqueness and overlap of differential probes deteced by DMH-v1 versus MMASS-v1, DMH-v2 versus MMASS-v2, and MMASS-v1 and MMASS-v2 versus MeDIP methods, respectively**. A greater amount of differential information could be obtained by MMASS compared to DMH and MeDIP using either enzyme sets.

To evaluate MMASS and DMH assays, McrBC [23,33,34] was used to restrict samples for the representation of unmethylated sequences; the combination of BstUI and HpaII (v1) [12,16,18-20] and the combination of AciI, HinP1I, HpyCH4IV and HpaII (v2) [23,33] were two sets of methylation-sensitive enzymes. Both sets of methylation-sensitive enzymes (v1 and v2) were able to interrogate more than 90% CGI probes on our array, with v2 as high as 98.81% (Table 1, 2). As seen from the MA and volcano plots, differential expression (M), statistical *B *value, and log_2_fold change of MMASS were much higher than DMH for both v1 and v2 enzyme sets (Figure 1). These data indicate that MMASS has a higher sensitivity than DMH. In addition, MMASS had a higher number of significant candidates (*B *value >0 as cutoff) than did DMH (Figure2-A, B): MMASS-v1 selected 531 differential candidates and MMASS-v2 had 512; whereas DMH-v1 selected 232 and DMH-v2 had 142. The overlap in candidates between DMH and MMASS using enzyme set v1 and set v2 was 144 and 114, respectively. Most of differential candidates detected in DMH were also selected by MMASS, but MMASS characterized an additional number of unique candidates (Figure2-A, B).

**Table 1:** Percent of coverage of CGI loci identified from the CGI library and CpGs in the human genome by each methylation-sensitive restriction enzyme.

*Enzymes*	*Recognition sequence*	*Percentage coverage of CGI locis*		*Percentage coverage of CpGs*	
		
		Number	Percent	CGI Library	Whole genome *
HpaII (BsiSI)	CCGG	8,145	87.48%	11.31%	8.60%
Hin6I (HinP1I)	GCGC	7,877	84.60%	11.85%	6.40%
AciI (SsiI)	CCGC	8,093	86.92%	13.10%	17.40%
HpyCH4IV	ACGT	5,261	56.50%	2.88%	6.60%
BstuI	CGCG	7,154	76.83%	9.14%	NA

* Percent coverage of CpGs in the whole genome cited from Schumacher *et al*., 2006, Nucleic Acids Reaserach, 34(2), 528-542, which was based on a representative 100 Kb of genomic DNA derived from the Chromosome 22 COMT region.

The total number of clones on the array was 9311 and the total number of CpGs in the clones on the array was 311052.

**Table 2:** Percent coverage of CGI loci identified from the CGI library using a combintion of enzymes.

*Enzymes*	*All Covered^1^*		*Overlapped Covered^2^*	
	
	Number	Percentage	Number	Percentage
HpaII (BsiSI) + BstuI (v1)	8,770	94.19%	6,529	70.12%
HpaII (BsiSI) + Hin6I (HinP1I) + AciI (SsiI) + HpyCH4IV (v2)	9,200	98.81%	3,885	41.72%

^1 ^All Covered means the clones cover at least one combined enzymes site.

^2 ^Overlapped Covered means clones cover all combined enzymes sites.

To validate the accuracy of the results from the two methods, we used bisulfite sequencing to confirm methylation statue difference in GES and 803 from the unique targets in MMASS-v2 and DMH-v2, and the common targets from both. For this, we randomly tested 25 of the unique MMASS-v2 targets (398 total), 13 of the unique DMH-v2 targets (28 total), and 17 of the candidates common to both (114 total), and obtained validation of 88.00% of the unique MMASS-v2 candidates, 75.00% of the unique DMH-v2, and 94.12% of the candidates common to both (Additional file 1, **Figure S3 (Top)**). Similar results also could be obtained from v1 set of enzymes that the true positive rates are 85.00% in MMASS-v1 unique and 96.55% in common. Thus, the data from the bisulfite sequencing validation indicates that both sensitivity and accuracy in MMASS was higher than the ones in DMH. Although it has been reported that the comparison of methylated to unmethylated DNA within sample in MMASS would amplify the fold change and *B *value, based on our validation data, the results are relatively reliable.

We also compared results between MMASS with MeDIP, for which, respectively, enrichment is based on methylation-sensitive and -dependent enzymes digestion, and on immunoprecipitation of methylated DNA. The MA and volcano plots of these two assays showed significantly higher differential expression (M), statistical *B *value, and fold change in MMASS for both v1 or v2 set of enzymes as compared to MeDIP, indicating that the sensitivity of MMASS assay is greater than MeDIP. Additionally, MMASS also identified more candidates showing differential expression compared to MeDIP (Figure2-C). Although enzyme digestion assays do have restriction-site limitations, since even with several enzymes combined the whole genome CGI cannot be covered (shown in Table 1), the MMASS assay still shows an overall better sensitivity than the other two, indicating it likely provides a better representation of the methylation status of target DNA.

We also evaluated the accuracy of the two assays by testing 27 candidates from unique to MMASS-v2 (420 total), 16 candidates unique to MeDIP (92 total), and 16 candidates shared by both (92 total) methods for bisulfite sequencing. The resulting data showed that the true positive rates were 85.71% for those unique to MMASS-v2, 85.71% for those unique to MeDIP, and 100.00% for those in common between the two methods (Additional file 1, **Figure S3 (bottom)**). Although the sensitivity of MMASS was much higher than MeDIP, the accuracy of both assays was similar.

Considering that MeDIP approach employed Klenow for amplification of purified methylated DNA fragment, it generally has lower amplification efficiency compared with the PCR based amplification employed in DMH and MMASS. Therefore we suspected that the lower sensitivity in MeDIP is due to the low abundance of tested DNA amplicon in MeDIP, which can not be detected by array. Under such perspective, we assessed the efficiency of immunoprecipitation. We used quantitative PCR analysis on 11 randomly selected differential methylated probes in the MGC-803 cell line that were validated by bisulfite sequencing as targets, and the results (Additional file 1, **Figure S4-A**) showed that MeDIP enriched the majority of the hypermethylated DNA fragments several fold relative to an equal amount of input DNA. We also assessed the enrichment level of these differential methylated clones in the Ges-1 cell line, and the methylation level of Ges-1 compared to MGC-803 was consistent with the results obtained using MMASS-v2 but was very low for MeDIP (*B *value <0) (Additional file 1, **Figure S4-B**). Based on these findings, the methylation difference of CGI between the two samples was likely due to the inability of MeDIP enrichment to be thoroughly using CGI array based technology. Given this, we decreased the threshold of the *B *value to obtain a larger number of differential candidates from the results of MeDIP. With the *B *value cutoff decreased to -1, -2, -3, -4, -5, and -6, the number of identified differential probes increased to 238, 308, 418, 615, 1000, and 1788. Respectively (Table 3); nevertheless, the relative overlap percentage of MeDIP with both MMASS-v1 and MMASS-v2 decreased gradually (Table 3). Furthermore, we validated the accuracy of newly covered unique differential probes from MeDIP with *B *value cutoff decreased through bisulfite sequencing. The results demonstrated that the true positive rate is 88.89% with -2 <*B *value < 0 which is similar with 85.71% of *B *value > 0, but it sharply descends to 52.17% as -4 <*B *value < -2 (Additional file 1, **Figure S5**). Therefore, a slight decrease in the *B *value can compensate for deficiencies the array allowing detection of more differential candidates without substantially affecting accuracy.

**Table 3:** Overlapped differential probes of MeDIP with MMASS-v1 and MMASS-v2 with a decreasing B value cutoff.

*B value (MeDIP)*	* > 0*	* > -1*	* > -2*	* > -3*	* > -4*	* > -5*	* > -6*
**Overlapped probes with MMASS-v1&MMASS-v2**	102	123	148	182	231	289	389
**Total differential probes**	184	238	308	418	615	1000	1788
**Percentage**	55.43%	51.68%	48.05%	43.54%	37.56%	28.90%	21.76%

## Discussion

Genomic profiling of methylated and unmethylated sequences using methylation-sensitive restriction enzyme digestion or 5'-methylcytosine antibody immunoprecipitation combined with hybridization to microarrays is a potentially powerful and expedient method. However, in contrast to work performed on expression microarray data, so far there has been no detailed assessment of the effects of different parameters or of enrichment assays on the overall results from these methods. Here, we optimized several important parameters to enhance the efficiency of enrichment, and elucidated the sensitivity and specificity of methylation-restriction enzyme-digestion-based methods and 5'-methylcytosine immunoprecipitation-based enrichment methods.

Our data showed that the sensitivity of DMH is less than that of MMASS, whereas DMH is complementary to MMASS. MMASS when used in combination with CGI array detection provided the best results for both the sensitivity and accuracy of the three different methylation profiling approaches when using a *B *value cutoff 0. The results when using methylated DNA as compared to unmethylated DNA in MMASS also increased the sensitivity of differential methylation detection, and this was primarily because both methylation sensitive and methylation-dependent enzymes are complementary in their ability to identify differential methylation levels in CGI. However, methylation-sensitive restriction enzymes were not able to interrogate every cytosine, and, even when using a combination of four enzymes, more than half of the CpG sites of the genome were missed. Thus, in MMASS, many of the differential CGIs ranked very low as candidates; these candidates, however, could be detected using MeDIP.

The above is likely due to the fact that 5'-methylcytosine antibody binds to methylated DNA throughout the entire genome, making it potentially a better method for detecting genome-wide methylation changes; however, it still has serious limitation in that, whereas it can easily detect methylated DNA where there are two or three methyl moieties per molecular, DNA with only one methyl group is insufficient for detection [26]. Methylated DNA enrichment could potentially aid in overcoming this issue, and our assessment of MeDIP did illustrate that such low copy CGIs could be enriched through immunoprecipitaton, they were poorly detected by the CGI array. Using a slightly lower *B *value cut-off did compensate for this, but lowering the cutoff too far had a drastic negative impact on specificity. Thus, only with very careful adjustment of specific parameters, could MeDIP achieve similar sensitivity and specificity to MMASS. It should also be noted that our study was performed for CpG islands, hence in CpG-poor regions the performance of the compared methods might differ.

This last finding indicated that, because such care is needed in setting the parameters for MeDIP to obtain good sensitivity without extensive loss of accuracy, that use of more sensitive detection technology such as next generation sequencing (NGS, under the platforms like Illumina Genome Analyzer, Roche/454 FLX, and Applied Biosystems SOLiD™system) [35,36], rather than array technology would make this an excellent method for getting at the methylation status of the entire genome. Genome-scale methylation profiling with bisulfite sequencing has been successfully performed in *Arabidopsis *and mammalian cell line [9,11,37]. Additionally, restriction enzymes have been combined with direct sequencing to determine global methylation patterns in human brain DNA [38]. With the development of high throughput sequencing, it is expected that whole genome methylome sequencing will become an even more effective strategy for whole epigenomic analysis. On the other hand, it should also be noted that although NGS technology with platform such as Illumina Genome Analyzer is not strictly restricted by abundance of DNA fragments and is able to provide whole genome methylation profile at single-nucleotide base resolution, currently the high cost and high computational load of bioinformatics analysis make it still of limited application. Microarray-based technology as we utilized in this work technically requires high abundance of DNA fragments to ensure high signal intensity needed for array and can not reach the resolution of single-nucleotide base, while it has been well-established with low cost and mature bioinformatics strategies and is especially applicable to detect methylation profile of specific regions of interest (*e.g*. CpG islands). Thus, NGS and microarray DNA methylation profiling methods are expected to co-exist to fulfill the demands of different researches in future [39]. The enrichment methods we evaluated in our work could be employed to enrich specific methylated genomic regions of interest for both microarray technology and NGS technology to investigate DNA methylation profiling.

## Conclusions

Our results show that assays based on methylation-sensitive enzyme digestion and those based on immunoprecipitation detected different methylated DNA fragments, indicating that they are complementary in their relative ability to detect methylation differences. Our study provides the first comprehensive evaluation for widely used methodologies for methylated DNA enrichment, and could be helpful for developing a cost effective approach for DNA methylation profiling.

## Methods

### CGI Library, sequencing and CGI array construction

The CGI Library was obtained from the Wellcome Trust Sanger Institute (Cambridge, UK). The library preparation was as described [40]. Library aliquots were grown in LB media plus ampicillin and plated on LB agar plates plus ampicillin. 17,606 individual clones were picked into 96-well plates containing 600 ul of LB plus ampicillin. Clones were grown in culture overnight at 37°C with shaking, followed by 50 ul of culture diluted with glycerol to a final concentration of 30% as stock solution kept at -80°C for later use. The remaining culture was used for plasmid DNA purification. Plasmid DNA was bi-directionally sequenced on MegaBACE DNA sequencer at the Beijing Genomics Institute, and BLAST was used to map the resulting sequences to the human genome (NIH 36.1). We also collected 6,445 unique clones from the CpG Island Tagging Project data of Wellcome Trust Sanger Institute http://www.sanger.ac.uk/HGP/cgi.shtml. Unique clones from two batches that overlapped with computational predictions of the whole genome CGI were selected and amplified by PCR for CGI array construction [41]. All purified PCR products from 9,223 CGI clones were sent to the CapitalBio Company for array spotting. Each clone was spotted in triplicate, and the whole array was composed of 48 blocks. In addition to CGI clones, external DNA controls, hexachloro-fluorescein (HEX), and Alien PCR product were placed in the first line of each block and negative controls such as DMSO, empty, and blank wells were also included in the array.

### Target probe preparation

Based on different combination of methylation-sensitive enzyme digestion, DMH-v1 and DMH-v2 methods were employed to enrich methylated DNA fragments for between-sample comparison [19], whereas MMASS-v1 and MMASS-v2 methods used methylation-sensitive and methylation-dependent enzyme digestion for within-sample comparison [23]. In both DMH and MMASS methods, 2 ug of genomic DNA were digested with MseI overnight at 37°C. The digested DNA was then ligated to the linkers H-14 (5'-tactccctcggata-3') and H-24 (5'-aggcaactgtgctatccgagggag-3') after purification using a Qiaquick PCR purification column (Qiagen, Germany). For enrichment of methylated DNA fraction (common in DMH and MMASS), half of the sample was digested with either a combination of BstUI and HpaII (v1) or a combination of AciI, HinP1I, HpyCH4IV, and HpaII (v2). For enrichment of the unmethylated DNA fraction in MMASS, half of the sample was digested with McrBC. PCR amplification of each enriched DNA fragment was performed in a 300 ul volume mixture comprised of the restriction DNA product, 10× ThermoPol Buffer, 2.5 uM H-24 primer, 0.2 mM dNTP mixture, and 12 U Deep Vent(exo-) DNA polymerase (New England Biolabs, USA). The amplification conditions were 5 min at 72°C to fill in the protruding ends of the ligated DNA fragments, followed by 20 cycles (1 min at 97°C and 3 min at 72°C), with a final extension for 10 min at 72°C. Ten ul of the PCR product underwent electrophoresis on a 1.5% agarose gel, with a smear between 0.2 and 2 kb indicating successful procedure performance during enrichment, as described previously [19]. MeDIP (5'-methylcytosine antibody) was employed to immunoprecipitate methylated DNA. 6.5 ug of genomic DNA underwent sonication into random fragments ranging in size from 200 to 1,000 bp. The performance procedures of MeDIP were similar to those as described in the work of Weber *et al *[25]. Briefly, sonicated DNA was denatured at 95°C for 10 min, then immunoprecipitated with 10 ul of monoclonal antibody against 5-methylcytosine (Merk, USA) to a final volume of 500 ul of IP buffer (10 mM sodium phosphate (pH 7.0), 140 mM NaCl, 0.05% Triton X-100) for 2 and 12 h at 4°C, respectively. Then the mixture was incubated with 30 ul of Dynabeads with M-280 sheep antibody to mouse IgG (Dynal Biotech) for 1, 2, 4, and 6 h at 4°C, respectively and washed 3× with 700 ul of IP buffer. The sample was incubated with proteinase K for 3 h at 50°C, and the methylated DNA was recovered by phenol-chroloform extraction followed by ethanol precipitation.

### CGI array hybridization and microarray data processing

For DMH and MMASS, 600 ng of each amplicon was labeled with Cy3/Cy5-dCTP (Amersham, USA) (0.24 mM of each dATP, dGTP, dTTP, 0.12 mM of dCTP and 0.12 mM of Cy3-dCTP or Cy5-dCTP) by random priming. For MeDIP, 1.5 ug of sonicated input DNA and the product of MeDIP (about 400 ng) were labeled with Cy3/Cy5-dCTP. The Cy3- and Cy5-labeled probes were purified with QIAquick PCR purification kit (Qiagen, Germany), mixed with yeast tRNA (20 ug) and Cot1 (10 ug), and reduced to a volume of 18.4 ul via speed vacuum. After denaturation (5 min at 95°C), hybridization buffer pre-warmed to 42°C (40 ul of formamide (Sigma, USA), 12 ul of 20× SSC (Amersham, USA), 1.6 ul of 10% SDS (Amersham, USA), and 8 ul of 50× Danhart's (Amersham, USA)) was added into the probe mixture to a final volume of 80 ul. Hybridization of the CGI array was performed under a cover slip in a humidified chamber fixed in a BioMix II hybridization machine (CapitalBio, China) at 42°C for 17 h. The array was washed two times in 2× saline sodium citrate and 0.2% SDS at 42°C for 5 min and once in 0.1× saline sodium citrate at room temperature. The slides were dried by centrifugation at 800 rpm for 5 min and scanned immediately with LuxScan 10 K scanner (CapitalBio, China). Image analysis was performed with LuxScan 10 K software (CapitalBio, China) and the raw data was exported as lsr file for subsequent data analysis.

The median average intensity of foreground and background was extracted from the lsr files. If a spot intensity was zero or negative after background subtraction, it was set at half of the minimum positive corrected intensities in the array [42]. We performed normalization using the spike probes that were previously shown to have a consistent log-ratio [43]. Then linear model and empirical Bayes smoothing analyses were combined to obtain the *B*-statistic (lods or *B*, *i.e*. the log-odds that the CpG island is differentially methylated) and fold change of each spot [44]. Significant candidates were selected with values of *B *> 0. All of above calculations were performed using limma http://bioinf.wehi.edu.au/limma/ package within the R environment http://cran.r-project.org/.

### Bisulfite genomic sequencing

Genomic DNA was treated with EZ DNA Methylation-Gold Kit™(Company, USA). All the CGIs of differential candidates were amplified with nested PCR. All primer information can be obtained in the supplementary information. The PCR products were gel extracted with QIAquick Gel Extraction Kit (Qiagen, Germany) and cloned into a TA cloning vector according to the manufacturer's instructions (pGEM-T-Easy cloning kit; Promega, USA). At least ten positive clones for each candidate were picked for sequencing. The methylation status of individual CpG sites was determined by comparison of the sequencing results with the original target sequence using CpGViewer software [45].

### Quantitative PCR validation

To assess the immunoprecipitation efficiency, primers were designed to amplify 90~150-bp fragments from differentially methylated regions which were not identified by MeDIP but identified by MMASS. Differentially methylated regions identified by both MeDIP and MMASS were chosen as controls. Equal amounts of methylated DNA enriched from immunoprecipitation and original input DNA were used as templates, respectively. Each plate of PCR reactions included β-Actin as an internal control, and at least three biological replicates were tested. In total, 11 CGI regions (8 cases and 3 controls) were tested for validation.

### Data Access

All the CpG island array data reported in this work is publicly available at Gene Expression Omnibus http://www.ncbi.nlm.nih.gov/geo/ with accession number GSE19974.

## Competing interests

The authors declare that they have no competing interests.

## Authors' contributions

ZSS and JW managed the project; LY and ZSS designed the experiments; LY and WD performed the experiments; KZ, XH and QZ analyzed the data; LY, KZ, JW and ZSS wrote the paper. All authors read and approved the final manuscript.

## Supplementary Material

Additional file 1 **Figure S1-S5 and Table S1-S2**. This file contains Figure S1-S5 and Table S1-S2. Click here for file
