# Supplementary material for: Systematic evaluation of genome-wide methylated DNA enrichment using a CpG island array
Source: BMC Genomics. 2011 Jan 6;12:10. doi: 10.1186/1471-2164-12-10 (PMC3023747; doi:10.1186/1471-2164-12-10)
Supplement: Additional file 1 — Figure S1-S5 and Table S1-S2. This file contains Figure S1-S5 and Table S1-S2. [file 1471-2164-12-10-S1.DOC]

**Figure S1-S5 and Table S1-S2**


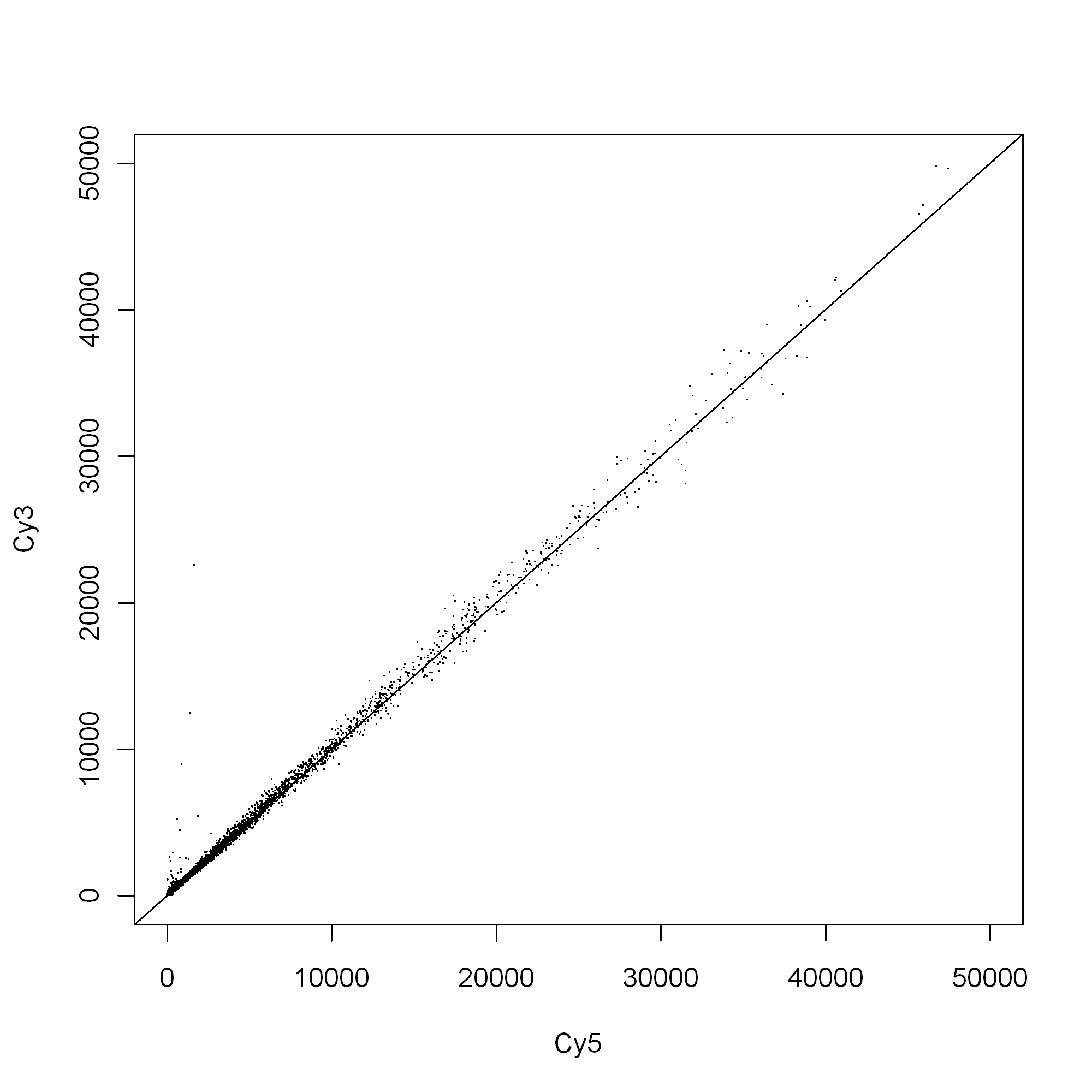


**Figure S1-A.** Intensity plot of human 9K CGI array after hybridization with 2 ug sonicated genomic DNA from MGC-803 cell line. Signal intensity of cy3 versus cy5 for all CGI probes is shown on the array. After normalization, the signal from the two channels shows a high correlation (Pearson Correlation Coefficient) of 0.9975.


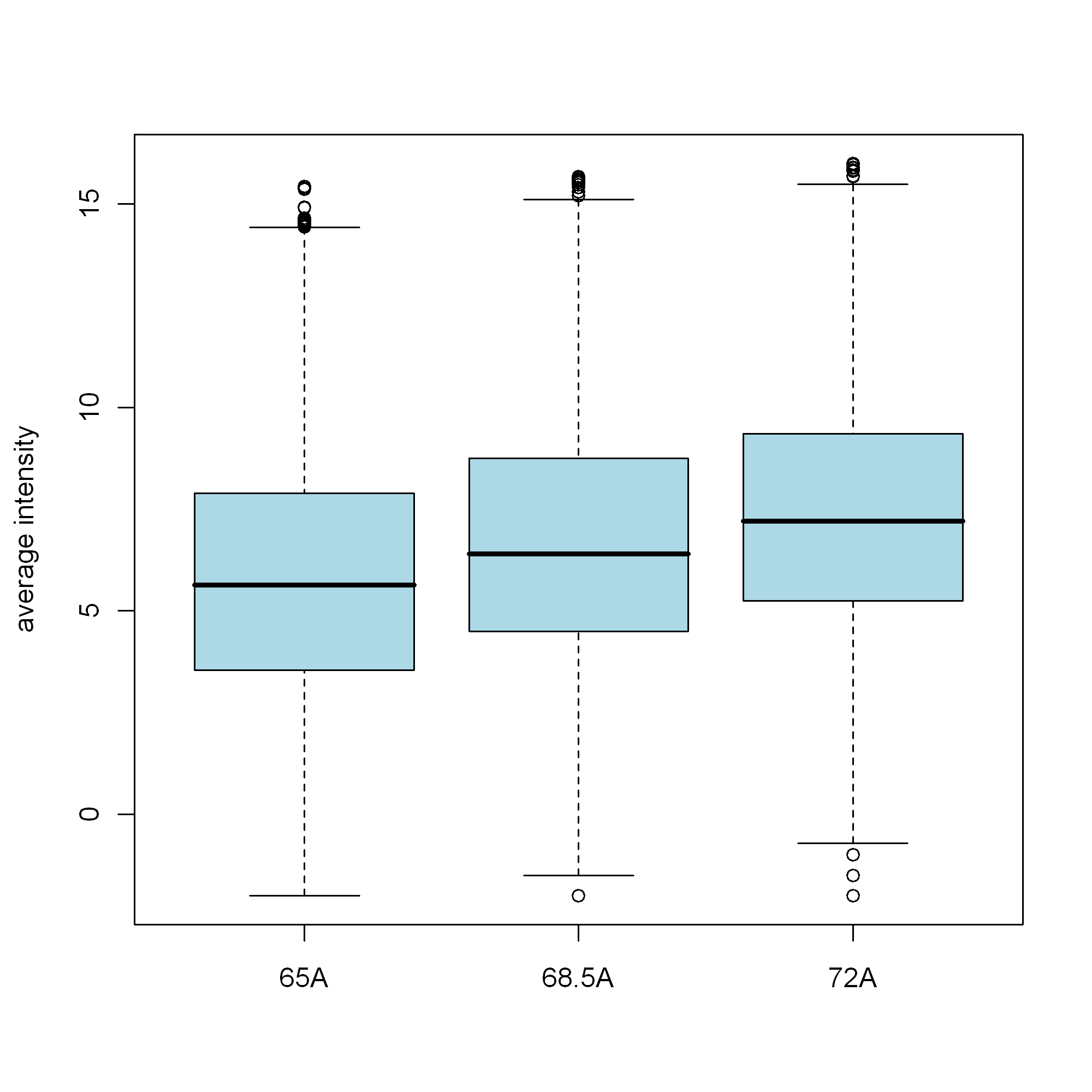


**Figure S1-B.** Box plot of CGI arrays after hybridization. The three boxes represent the total intensity of the CGI array after hybridization using the MMASS-v1 method in which an enzyme-digested product from genomic DNA from the MGC-803 cell line was amplified at three different annealing temperatures. The results show an increase in the total intensity with an increasing annealing temperature from 65°C to 72°C.


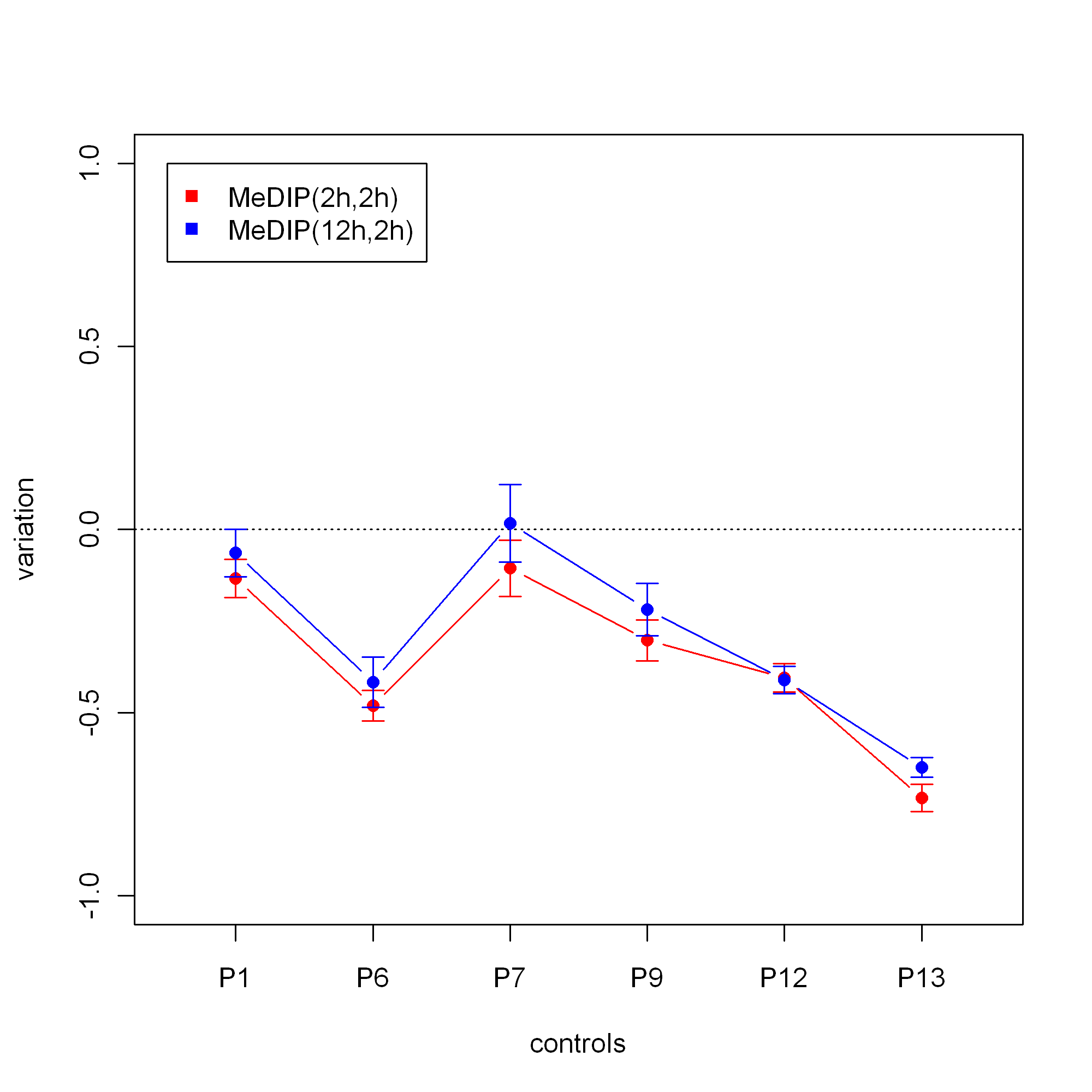


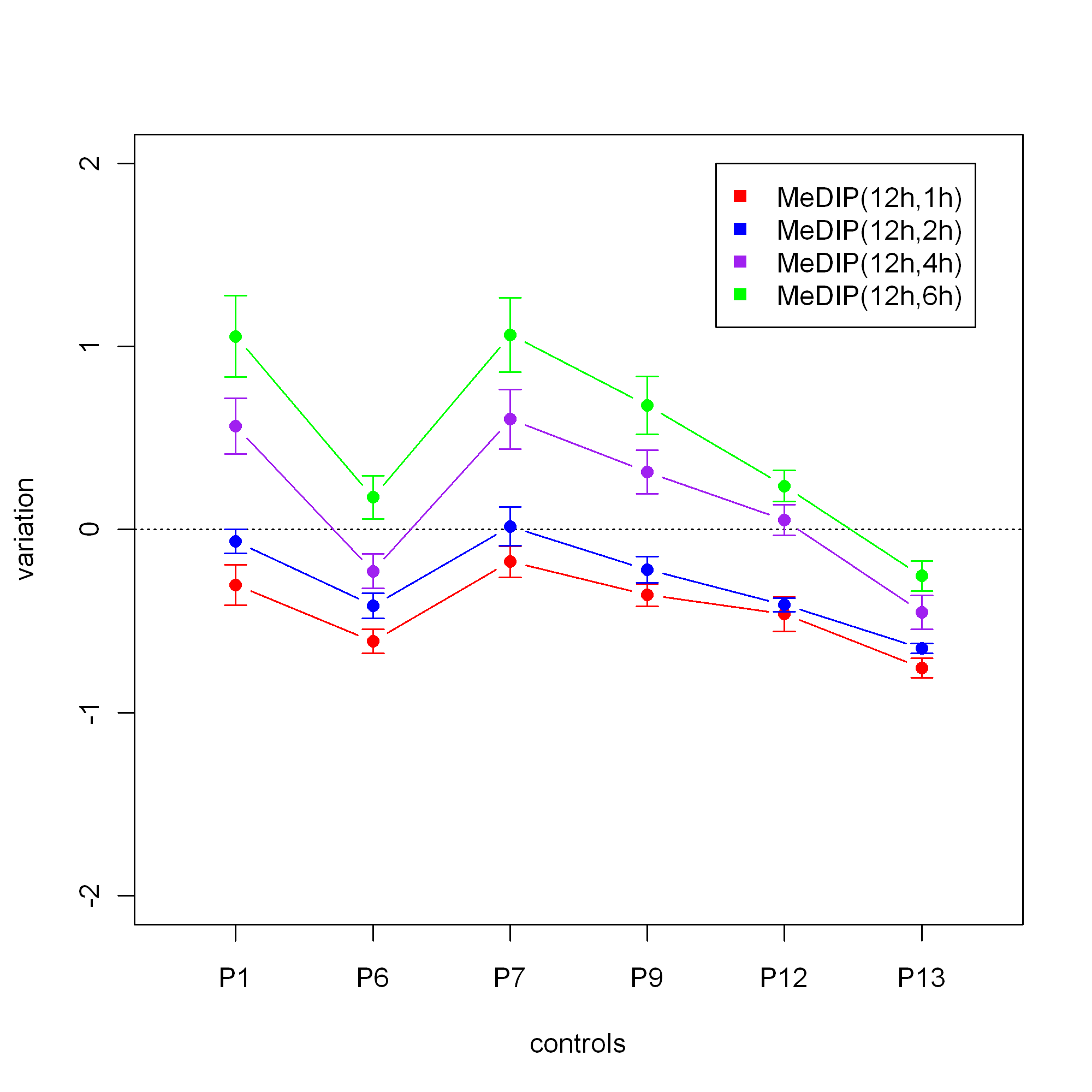


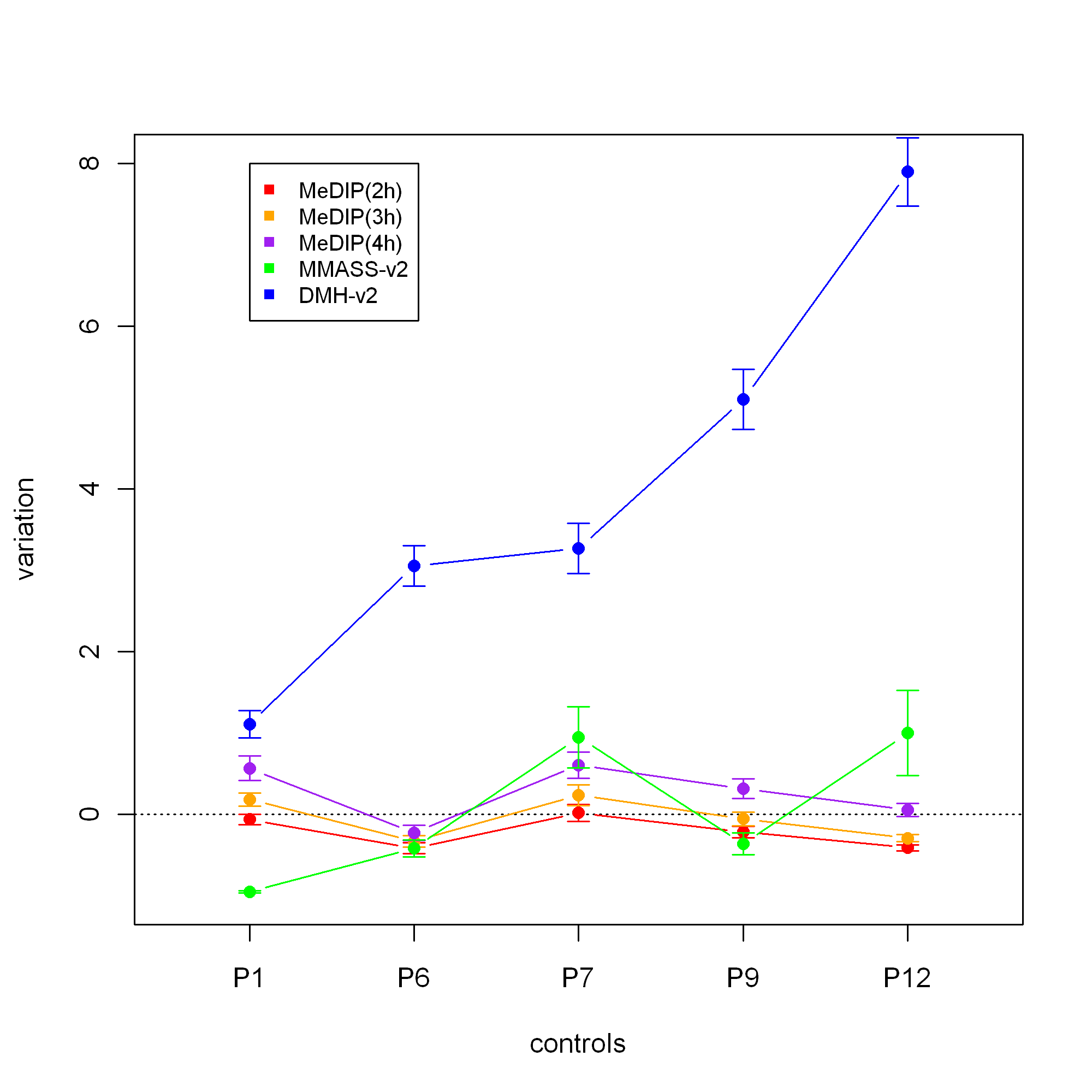


**Figure S2.** Deviation from the theoretical ratio of each external control after hybridization resulting from MeDIP with different antibody incubation times. (**Top**) The deviation of external controls decreased with a primary incubation time increasing from 2 hrs to 12 hrs. This indicated the binding efficiency of methylated external controls was improved if the primary antibody incubation time was increased. (**Middle**) Secondary antibody incubation time optimized as 1, 2, 4, and 6 hrs. The deviation of external controls decreased to minima with the secondary antibody incubation time from 2 to 4 hrs. Although the binding efficiency of methylated external controls was improved when the secondary antibody incubation time was increased, unspecific binding also rose correspondingly. (**Bottom**) The deviations of external controls resulting from each of the methods, including MeDIP with secondary antibody incubation for 2, 3, 4 hrs, MMASS-v2, and DMH-v2, respectively.

**Figure S3.** Bisulfite sequencing verification results. (**Top**) The verified positive rates of bisufite sequencing are 88.00% in MMASS-v2 uniqueness, 75.00% in DMH-v2 uniqueness, and 94.12% in common, respectively. (**Bottom**) The verified positive rates of bisufite sequencing are 85.71% in MMASS-v2 uniqueness, 85.71% in MeDIP uniqueness and 100% in common, respectively.

**Figure S4-A.** Evaluation of the efficiency of methylated DNA enrichment through quantitative PCR. All the selected clones were validated as hypermethylated in the MGC-803 cell line by bisulfite sequencing. The results of quantitative PCR show that MeDIP enriched all the methylated DNA fragments several fold relative to equal amounts of input DNA.

**Figure S4-B.** Validation of the methylation differences of several selected clones between Ges-1 and MGC-803 through quantitative PCR. These differentially methylated clones were identified in MMASS-v2 and verified with bisulfite sequencing, but ranked low in MeDIP. The results show that the methylation differences of Ges-1 compared to MGC-803 of these clones was significant and consistent with bisulfite sequencing.

**Figure S5.** Bisulfite sequencing verification results with B value in MeDIP decreased. The entire clones selected for bisulfite sequencing verification include: 27 candidates from MMASSv2 unique with B value more than 0, 16 candidates from MeDIP unique with B value more than 0, 18 candidates from MeDIP unique with B value between 0 and -2, and 23 candidates from MeDIP unique with B value between -2 and -4. The verified positive rates of bisufite sequencing are 85.71% in MMASS-v2 uniqueness (B value >0), 85.71% in MeDIP uniqueness (B value >0), 88.89% in MeDIP uniqueness (-2 < B value < 0) and 52.17% in MeDIP uniqueness (-4 < B value < -2), respectively. The ture positive rates were similar when B value in MeDIP decrease from 0 to -2, but sharply descends as B value decrease to -4.

**Table S1.** The theoretical ratio of methylated fragments compared to unmethylated fragments in each DNA external control from X and Y sets.

| ***M/UN*** | ***P1*** | ***P6*** | ***P7*** | ***P9*** | ***P12*** | ***P13*** | ***P14*** |
| --- | --- | --- | --- | --- | --- | --- | --- |
| **X** | 10:1 | 10:0 | 2:8 | 1:9 | 10:0 | 1:0 | 0:10 |
| **Y** | 2:8 | 10:0 | 10:0 | 10:0 | 1:9 | 1:0 | 0:10 |

**Table S2. The ratio of each DNA external control from X and Y sets after hybridization from each method.**

| ***Methods*** | ***Sets*** | ***P1*** | ***P6*** | ***P7*** | ***P9*** | ***P12*** | ***P13*** | ***P14*** |
| --- | --- | --- | --- | --- | --- | --- | --- | --- |
| **MeDIP(IP/Input)** | **X** | 40:11 | 4:1 | 4:5 | 2:5 | 4:1 | 4:1 | NA(0:2.5) |
|  | **Y** | 4:5 | 4:1 | 4:1 | 4:1 | 2:5 | 4:1 | NA(0:2.5) |
|  |  |  |  |  |  |  |  |  |
| **MMASS(M/UN)** | **X** | 10:1 | 10:0 | 1:4 | 1:9 | NA(10:0) | NA(1:0) | NA(0:10) |
|  | **Y** | 1:4 | 10:0 | 10:0 | 10:0 | 1:9 | 1:10 | NA(0:10) |
|  |  |  |  |  |  |  |  |  |
| **DMH(M/M)** | **Y/X** | 1:5 | 1:1 | 5:1 | 10:1 | 1:10 | 1:1 | NA(0:0) |
